# Macrophages reprogrammed by lung cancer microparticles promote tumor development via release of IL-1β

**DOI:** 10.1038/s41423-019-0313-2

**Published:** 2019-10-24

**Authors:** Jie Chen, Weiwei Sun, Huafeng Zhang, Jingwei Ma, Pingwei Xu, Yuandong Yu, Haiqing Fang, Li Zhou, Jiadi Lv, Jing Xie, Yuying Liu, Ke Tang, Bo Huang

**Affiliations:** 1 Department of Biochemistry and Molecular Biology, Tongji Medical College, Huazhong University of Science and Technology, 430030 Wuhan, China; 2 Department of Immunology, Institute of Basic Medical Sciences & State Key Laboratory of Medical Molecular Biology, Chinese Academy of Medical Sciences and Peking Union Medical College, 100005 Beijing, China; 3 Clinical Immunology Center, Chinese Academy of Medical Sciences, 100005 Beijing, China

**Keywords:** Tumor microparticles, Interleukin-1β, Macrophage, Inflammasome, Tumor-repopulating cells, Tumour immunology, Tumour immunology

## Abstract

Despite their mutual antagonism, inflammation and immunosuppression coexist in tumor microenvironments due to tumor and immune cell interactions, but the underlying mechanism remains unclear. Previously, we showed that tumor cell-derived microparticles induce an M2 phenotype characterized by immunosuppression in tumor-infiltrating macrophages. Here, we further showed that lung cancer microparticles (L-MPs) induce macrophages to release a key proinflammatory cytokine, IL-1β, thus promoting lung cancer development. The underlying mechanism involves the activation of TLR3 and the NLRP3 inflammasome by L-MPs. More importantly, tyrosine kinase inhibitor treatment-induced L-MPs also induce human macrophages to release IL-1β, leading to a tumor-promoting effect in a humanized mouse model. These findings demonstrated that in addition to their anti-inflammatory effect, L-MPs induce a proinflammatory phenotype in tumor-infiltrating macrophages, promoting the development of inflammatory and immunosuppressive tumor microenvironments.

## Introduction

Although immunosuppressive molecules and cells can inhibit inflammatory signals, inflammation and immunosuppression are always linked and coexist in tumor microenvironments, synergistically promoting tumor development.^1,2^ To date, the evolution of this coexistence in tumors remains incompletely understood. Cell death is a fundamental biological event that occurs during tumor development. Dying tumor cells may release a series of immunosuppressive molecules, such as cAMP, prostaglandins, indole-2,3-dioxygenase and arginase 1, to mediate immunosuppression,^3,4^ which also results in the release of inflammatory mediators, such as HMGB1 and HSP70, to promote inflammation.^5,6^ In addition to the above single molecules, apoptotic tumor cells may release apoptotic bodies, a type of vesicle with sizes ranging from 1 to 5 μm,^7^ which usually do not induce inflammation. Notably, dying tumor cells also release extracellular vesicles (EVs), a structure with a size between that of molecules and cells^8^. EVs can remodel the tumor microenvironment; however, their roles in the regulation of tumor inflammation remain poorly understood.^9^ Two types of EVs have been identified: exosomes and microvesicles (MVs).^8^ Exosomes are endosome-derived EVs (30–200 nm)^10,11^ that deliver information about proteins, mRNAs, and microRNAs to recipient cells. MVs are plasma membrane-derived shedding vesicles with sizes ranging from 0.1 to 1 μm.^8,12^ In response to various stimuli or apoptotic signals, cells reorganize their cytoskeleton, leading to encapsulation of the cytosolic contents within the cellular membrane to form vesicles that are subsequently released into extracellular spaces. In some studies, microparticles (MPs) are also known as microvesicles.^8,9,12,13^ We have shown that tumor cell–derived MPs can be readily taken up by DCs, leading to DC maturation and presentation of tumor antigens.^14,15^ After these MPs are taken up by macrophages, they can induce the development of macrophages with an M2 phenotype.^16^ However, the role of tumor cell-derived MPs in tumor inflammation and immunosuppression remains unclear.

Lung cancer is the leading cause of cancer-related death worldwide.^17^ Although tyrosine kinase-based targeted therapy can effectively interfere with oncogenic signaling, leading to substantial tumor cell death, the remnant tumor cells inevitably acquire drug resistance in half a year to 1 year.^18^ Notably, these drug-resistant cells show exacerbated malignant traits and lead to rapid clinical relapse and disease progression.^19^ Although the underlying mechanism is unknown, high levels of tumor cell death induced by targeted therapy inevitably result in high levels of MPs in the tumor microenvironment, suggesting that the MPs are involved in the above tumor progression. IL-1β, a key proinflammatory cytokine, is initially located in the cytosol as a precursor and is released into the extracellular space in its active form after cleavage by activated caspase-1.^20,21^ Importantly, IL-1β is commonly present in the tumor microenvironment and plays a tumor-promoting role.^22^ Previously, we found that tumor cell-derived MPs induce the polarization of macrophages toward the M2 phenotype, leading to subsequent anti-inflammatory and immunosuppressive effects.^16^ Unexpectedly, in the present study, we found that in addition to the induction of the M2 phenotype, lung cancer cell-derived MPs (L-MPs) can induce macrophages to release IL-1β via activation of the inflammasome pathway, thus remodeling tumor inflammation and immunosuppression and leading to lung cancer development.

## Methods

### Mice and cell lines

Six- to eight-week-old female BALB/c and C57BL/6 mice were purchased from the Center of Medical Experimental Animals of Hubei Province (Wuhan, China) for studies approved by the Animal Care and Use Committee of Tongji Medical College. Four-week-old female B-NSG mice were purchased from Beijing Biocytogen. Mouse Lewis lung cancer; Hepa1-6 and HepG2 hepatocarcinoma; B16 melanoma; human H460, A549, and HCC827 lung cancer and A375 melanoma tumor cell lines were purchased from the China Center for Type Culture Collection (Wuhan, China) and cultured according to the guidelines given.

### Preparation of T-MPs and blood cell-derived MPs

Tumor cells or blood cells derived from mice or healthy donors were exposed to ultraviolet radiation (300 J/m^2^) for 1 h, and 24 h later, the supernatants were used for MP isolation as described previously.^14,23^ Briefly, the supernatants were centrifuged at 200 × *g* for 10 min to remove whole cells and then centrifuged at 2700 × *g* for 10 min and 14,000 × *g* for 2 min to remove debris. The supernatants were further centrifuged at 14,000 × *g* for 1 h to pellet the MPs. We used a Fiberlite™ F15-6 × 100 y rotor (Thermo Scientific™). The MPs were washed three times and suspended in culture medium for the following experiments. The MPs were quantified and characterized using a nanoparticle tracking analysis (NTA) system (Nanosight NS300, Malvern) as described previously.^24^

### Preparation of mouse bone marrow- or human PBMC-derived macrophages

Bone marrow cells isolated from the femurs of mice were cultured for 5 days in the presence of 20 ng/ml recombinant mouse macrophage colony-stimulating factor (M-CSF) (PeproTech, Rocky Hill, NJ, USA) in complete RPMI 1640 medium containing 10% fetal bovine serum (FBS), 10 mM glucose and 2 mM l-glutamine.

Human PBMCs were isolated from human peripheral blood using density gradient separation. Monocytes were purified with human CD14 MicroBeads (Miltenyi Biotec, Bergisch Gladbach, Germany) and then cultured in complete RPMI 1640 medium containing 20 ng/ml recombinant human M-CSF (PeproTech) for the induction of macrophages. After 7 days, human macrophages were harvested and stimulated with T-MPs.

### Tumorigenic cell culture in soft three-dimensional fibrin gels

Salmon fibrinogen and thrombin were purchased from Sea Run Holdings (Kennebunkport, ME, USA). The detailed methods were previously described.^25^ Briefly, tumor cells were detached from the standard culture conditions and suspended in DMEM (10% FBS), and the cell density was adjusted to 10^4^ cells/ml. Fibrinogen was diluted to 2 mg/ml with T7 buffer (pH 7.4, 50 mM Tris, 150 mM NaCl). A 1:1 fibrinogen and cell solution mixture was made and had 1 mg/ml fibrinogen and 5000 cells/ml in the mixture. A total of 250 μl of the cell/fibrinogen mixtures was seeded into each well of a 24-well plate and mixed well with pre-added 5 μl thrombin (0.1 U/μl) for culture under 37 °C conditions.

### Gene silencing assay

siRNAs and negative control siRNAs were purchased from RiboBio (Guangzhou, China). siRNA (50 nM) was transfected into macrophages using Lipofectamine RNAiMax (Thermo Fisher Scientific, Waltham, MA, USA) according to the manufacturer’s instruction. The siRNA sequences are shown in Supplementary Table 1.

### Real-time PCR

Total RNA (1 μg) was extracted from cells or tumor tissues with TRIzol reagent (Thermo Fisher Scientific) and reverse-transcribed into cDNA by using the ReverTra Ace Kit (Toyobo, Osaka, Japan). The cDNA was amplified via THUNDERBIRD SYBR qPCR Mix (Toyobo) on a Bio-Rad CFX Connect Real-Time PCR System (Bio-Rad). The mRNA levels were normalized to that of β-actin. The primer sequences are shown in Supplementary Table 2.

### Western blot analysis

Cell lysates and prestained molecular weight markers were separated by SDS-PAGE, followed by transfer onto nitrocellulose membranes. The membranes were blocked in Tris-buffered saline with 0.1% Tween-20 containing 5% bovine serum albumin (BSA) and probed with a specific antibody overnight at 4 °C. Antibodies against pro-IL-1β, NF-κB, phospho-NF-κB, phospho-IKKα/β, phospho-IΚBα, JNK, phospho-JNK, p44/42 MAPK (Erk1/2), phospho-p44/42 MAPK (Erk1/2), p38 MAPK and phospho-p38 MAPK were obtained from Cell Signaling Technology (Danvers, MA, USA). Antibodies against caspase-1 p10 were obtained from Santa Cruz Biotechnology (Santa Cruz, CA, USA). Antibodies against V0a2 and V0a3 were obtained from Thermo Fisher Scientific. Antibodies against β-actin were obtained from Proteintech (Wuhan, China). The membranes were washed three times and incubated with horseradish peroxidase-conjugated secondary antibodies. The immunoreactivity was visualized by enhanced chemiluminescence according to the manufacturer’s protocol.

For MP samples, MP lysates (30 μg of total protein) were separated by SDS-PAGE and transferred onto nitrocellulose membranes for western blot detection. The protein quantity of the samples was assessed by using a BCA assay kit according to the manufacturer’s instructions (Thermo Fisher). Briefly, solutions A and B were mixed at a ratio of 1:50, and as the substrate, BSA standards at various dilutions or samples were added to the solution. Absorption was detected at 562 nm, and the protein concentration was calculated through a standard curve.

### Immunofluorescence staining

For immunofluorescence staining, cells were seeded in confocal dishes for 24 h with or without L-MP treatment. The cells were fixed with 4% paraformaldehyde in PBS pH 7.4 for 10 min at room temperature and then permeabilized with 0.5% Triton X-100 in PBS for 10 min. After the cells were blocked with 2% BSA in PBS containing 0.1% Tween-20 for 30 min, they were incubated with anti-V0a2 or anti-V0a3 antibody (Thermo Fisher Scientific). In some cases, tumor frozen sections were stained with anti-F4/80 or anti-IL-1β antibody (Thermo Fisher Scientific) in 2% BSA in PBS containing 0.1% Tween-20 overnight at 4 °C. After the samples were washed and stained with secondary antibody for 1 h at room temperature, the nucleus was stained with DAPI (4′, 6-diamidino-2-phenylindole) (2 μg/ml). The merged figures were analyzed by a two-photon fluorescence microscope.

### Flow cytometric analysis

Cell surfaces were stained with PE-Cy5 conjugated anti-CD11b and PE conjugated anti-F4/80 (Thermo Fisher Scientific). For intracellular staining, live cells were first fixed with the fixation buffer, then treated with permeabilization buffer and stained with APC conjugated anti-IL-1β (Thermo Fisher Scientific). Data were acquired on an Accuri C6 system (BD Biosciences, San Jose, CA, USA) and analyzed with FlowJo software.

### Measurement of the intracellular calcium concentration

Cells were incubated with 5 μM Fluo-4 AM (Thermo Fisher Scientific) in PBS. The calcium concentration was determined by measuring the Fluo-4 mean fluorescence intensity with ionomycin stimulation (Abcam, Cambridge, MA, USA) and acquired on an Accuri C6 system (BD Biosciences) with a low speed.

### Hematopoietic stem cell isolation

Fresh human umbilical cord blood was obtained from Tongji Hospital, according to guidelines approved by the ethics boards and the Clinical Research Committee at Tongji Hospital. The hematopoietic stem cells (HSCs) were isolated as previously described.^26^ Briefly, umbilical cord blood mononuclear cells were separated via Ficoll-Hypaque density gradients. CD34^+^ HSCs were isolated by using a direct CD34 Progenitor Cell Isolation Kit (Miltenyi Biotec). More than 95% of the CD34^+^ cells were positively selected after two rounds of enrichment. The HSCs were transplanted within 24 h after isolation.

### Transplantation of CD34^+^ cells into B-NSG mice

All animal work was conducted in accordance with a protocol approved by the Institutional Animal Care and Use Committee at Tongji Medical College. Female B-NSG mice (4 weeks old) were subjected to 200 cGy of total body irradiation 12 h before injection with 3 × 10^5^ HSCs in 0.2 ml of medium via the tail vein. At 4 weeks after HSC transplantation, plasmids in which human *M-CSF* was cloned into the GV208 vector (Genechem, Shanghai, China) were injected into the mice. Plasmid DNA was extracted with an EndoFree Plasmid Maxi Kit (QIAGEN, Stockach, Germany). For hydrodynamic injection, 50 μg of plasmid in 1.8 ml of PBS was injected into humanized mice within 7 s using a 27-gauge needle once a week. At 6 weeks after HSC transplantation, 5 × 10^6^ HCC827 cells were injected into the right thigh muscle of the mice. For antibody treatment, the mice were i.p. injected with 3 mg/kg of purified anti-human IL-1β neutralizing antibody (Thermo Fisher Scientific) 8 weeks after HSC transplantation twice weekly. The day after the neutralizing IL-1β antibody injection, the mice were intratumorally injected with L-MPs once every 2 days. 23 days after the tumor inoculation, the mice were sacrificed, and the tumor weight was measured. Tumor sections were labeled by immunofluorescence to indicate the distribution of macrophages (CD68 green) and IL-1β (navy blue). The distribution of HCC827-MPs (red) was also recorded. Cell nuclei were stained with DAPI.

### Statistical analysis

The results are presented as the mean ± SEM, and statistical significance was assessed via an unpaired two-tailed Student’s *t* test by using GraphPad 6.0 software. The log-rank (Mantel–Cox) test was used to analyze the long-term survival curve. A P-value < 0.05 was considered statistically significant.

## Results

### M2-like macrophages induced by L-MPs upregulated IL-1β expression

**Fig. 1:**
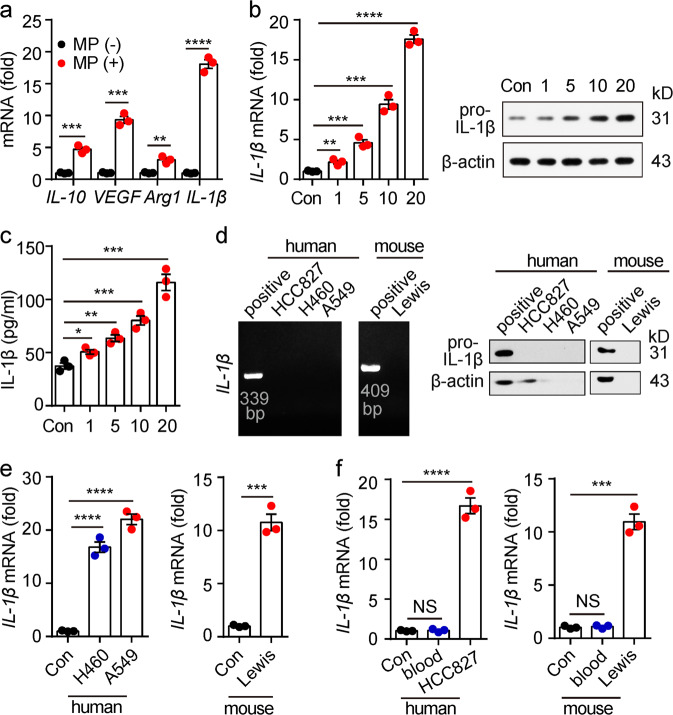
L-MPs induced macrophages to upregulate IL-1β expression. **a** Human PBMC-derived macrophages were treated with HCC827-MPs at a ratio of 1:20 (macrophages: MPs). After 12 h, the cells were collected, and RNA was extracted for real-time PCR analysis of *IL-10*, *arginase 1* (*Arg1*), *VEGF* and *IL-1β*. **b**, **c** Human PBMC-derived macrophages were treated with HCC827-MPs at different ratios (cell:MPs, 1:1, 1:5, 1:10, 1:20). RNA, protein and cultured medium were collected after 12, 24, or 72 h of treatment, respectively. Then, the IL-1β expression was analyzed by real-time PCR, western blots (**b**) or ELISAs (**c**). **d** *IL-1β* mRNA or pro-IL-1β expression of HCC827-MPs, H460-MPs, A549-MPs and Lewis-MPs was analyzed by RT-PCR (left) or western blot (right) analyses. Human PBMC-derived macrophages treated with HCC827-MPs were used as positive controls for A549, HCC827, and H460-MPs. Mouse BMDMs treated with Lewis-MPs were used as a positive control for Lewis-MPs. **e** Human PBMC-derived macrophages were treated with H460-MPs or A549-MPs for 12 h (left). Mouse BMDMs were treated with Lewis-MPs for 12 h (right). Then, the *IL-1β* mRNA level was analyzed by real-time PCR. **f** Human PBMC-derived macrophages were treated with healthy human blood cell-derived MPs at a ratio of 1:20 (cell:MPs). *IL-1β* mRNA levels were analyzed by real-time PCR (left). BMDMs were treated with wild-type mouse (C57BL/6) blood cell-derived MPs at a ratio of 1:20, and then, the *IL-1β* mRNA level was analyzed by real-time PCR (right). Error bars indicate the mean ± SEM; n = 3 independent experiments. *P < 0.05, **P < 0.01, ***P < 0.001, ****P < 0.0001

Macrophages are the main immune cell type in tumor microenvironments, where they exert an inhibitory or promotional effect on tumors, depending on their M1 or M2 phenotype. Previously, we showed that tumor cell-derived MPs induce an immunosuppressive M2 phenotype that promotes tumor progression in macrophages.^16^ Consistent with these results, the treatment of macrophages with HCC827 (a human lung cancer cell line)-derived MPs (L-MPs) resulted in the upregulation of *VEGF*, *IL-10* and *arginase 1* expression and the downregulation of *IL-12*, *iNOS* and *TNF-α* expression (Fig. 1a, supplementary Fig. 1). In addition, the expression levels of CD163 and CD206, two surface markers of M2-type macrophages, were also upregulated in L-MP-treated macrophages (Supplementary Fig. 2). These treated macrophages could inhibit T cell activation, suggesting they display an M2 phenotype that has an anti-inflammatory role.^27^ Notably, L-MPs could induce a switch in M1 macrophages toward the M2 phenotype, as shown by the finding that LPS-conditioned M1 macrophages upregulated *VEGF*, *IL-10* and *arginase 1* (supplementary Fig. 3). To our surprise, the L-MP-treated macrophages also upregulated the mRNA expression of *IL-1β*. Consistent with this finding, pro-IL-1β and the cleaved mature IL-1β were also upregulated at the protein levels by hL-MPs in a dose-dependent manner, as shown by real-time PCR, western blots and ELISAs (Fig. 1b, c). This result was not ascribable to the inclusion of IL-1β in the L-MPs since *IL-1β* mRNA and protein were not detected in L-MPs (Fig. 1d). Notably, L-MPs from other lung cancer cell lines (murine Lewis or human A549 and H460 cells lines) also induced macrophages to upregulate *IL-1β*, but the MPs isolated from other tumor cells, healthy murine cells or human blood cells did not have such an effect (Fig. 1e, f, supplementary Fig. 4). Using a NTA system, we found that HCC827-MPs and Lewis-MPs were ~161 and 152 nm in size, respectively, consistent with the size of MPs, which show a range of 0.1–1 μm. These L-MPs expressed surface membrane molecule major histocompatibility complex class I, and HCC827-MPs expressed the cytoplastic proteins tubulin and heat shock protein 70 (HSP70); however, serum protein apolipoprotein B (ALB) and nuclear protein histone 2B showed very low expression in L-MPs (Supplementary Fig. 5), suggesting that these vesicles belong to MPs but are not apoptotic bodies or serum protein complexes. Together, these data suggest that L-MPs may have certain unique trait(s) promoting macrophages to produce IL-1β.

### TLR3 activation is required for IL-1β induction by L-MPs

**Fig. 2:**
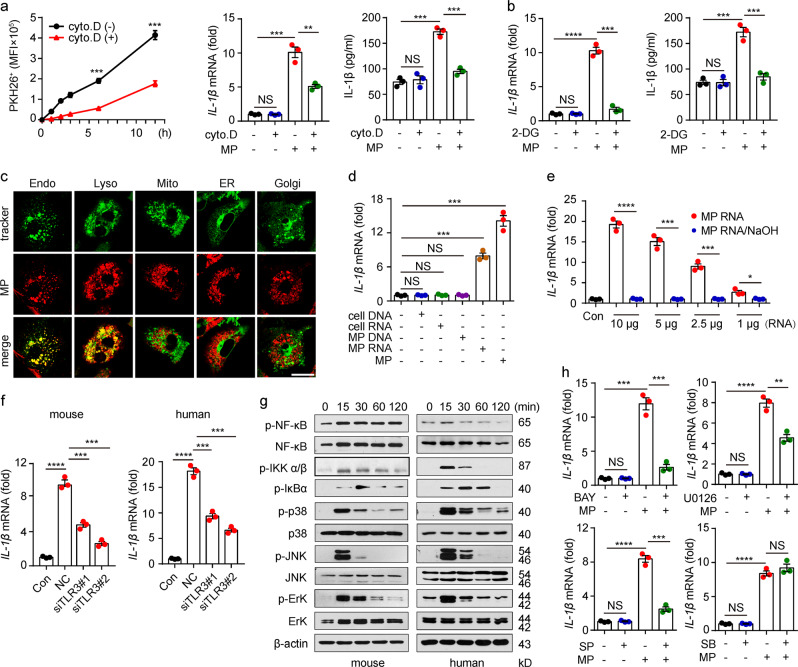
TLR3 activation is required for IL-1β induction by L-MPs. **a** Mouse BMDMs were treated with PKH26-stained Lewis-MPs with or without cytochalasin D (1 μg/ml) for 30 min. Then, the PKH26 fluorescence intensity of macrophages was measured by flow cytometry at different times (left), and the IL-1β expression level was analyzed by real-time PCR (middle) and western blot analyses (right). **b** Mouse BMDMs were pretreated with or without 2-DG (5 mM) for 30 min. Then, the BMDMs were treated with Lewis-MPs. *IL-1β* mRNA levels were analyzed by real-time PCR, and mature IL-1β in the supernatant was detected by ELISA after 12 and 72 h. **c** Mouse BMDMs were incubated with PKH26-labeled Lewis-MPs. After 12 h, the BMDMs were stained with live cell molecular probes, including mitochondria, ER, Golgi, endosome or lysosome trackers. Then, the MP location was observed under a two-photon confocal microscope. Scale bar, 20 µm. **d** Mouse BMDMs were treated with DNA (1 μg) or RNA (2.5 μg) extracted from Lewis cells or Lewis-MPs for 12 h, and then, the *IL-1β* mRNA level was analyzed by real-time PCR (left). **e** Mouse BMDMs were treated with Lewis-MP-derived RNA (10, 5, 2.5, or 1 μg) only or Lewis-MP-derived RNA pretreated with NaOH (0.5 M) for 12 h, and then, the *IL-1β* mRNA level was analyzed by real-time PCR (right). **f** Mouse BMDMs or human PBMC-derived macrophages were transfected with TLR3 siRNAs and then treated with Lewis-MPs or HCC827-MPs. After 12 h, the *IL-1β* mRNA level was analyzed by real-time PCR. **g** Mouse BMDMs (left) or human PBMC-derived macrophages (right) were treated with Lewis-MPs or HCC827-MPs, respectively. Then, the cells were collected, and the phosphorylation of NF-κB, IKK-α/β, IκBα, p38, JNK, and ERK was detected by western blot analyses at different times. **h** BMDMs were treated with Lewis-MPs in the presence or absence of NF-κB or MAPK inhibitors, including BAY 11-7085 (BAY), SB203580 (SB), U0126, or SP600125 (SP). After 12 h, the *IL-1β* mRNA level was analyzed by real-time PCR. Error bars indicate the mean ± SEM; *n* = 3 independent experiments. *P < 0.05, **P < 0.01, ***P < 0.001, ****P < 0.0001

Next, we investigated the molecular mechanism underlying the induction of IL-1β by L-MPs. Macrophages are professional phagocytes that take up extracellular materials via phagocytosis. Cytochalasin D, an endocytosis inhibitor that blocks the uptake of L-MPs, led to disruption of the above IL-1β induction (Fig. 2a). Endocytosis is an ATP-consuming process. Consistent with this fact, 2-deoxy-d-glucose-mediated inhibition of ATP generation also abrogated IL-1β expression (Fig. 2b), suggesting that the endocytosis of L-MPs is required for macrophages to upregulate IL-1β expression. Consistently, fluorescent staining showed that L-MPs were localized in endosomes and lysosomes but not in the Golgi apparatus, endoplasmic reticulum or mitochondria (Fig. 2c). IL-1β upregulation is commonly mediated through the activation of Toll-like receptor(s).^28,29^ Notably, TLR3, TLR7, TLR8 and TLR9 are localized on the membranes of endosomes,^29,30^ prompting us to hypothesize that L-MPs enter the endosomes of macrophages where they activate TLRs for IL-1β induction. Intriguingly, if we extracted RNA and DNA from L-MPs and used them to stimulate macrophages, we found that RNA, but not DNA fragments, from L-MPs induced *IL-1β* upregulation (Fig. 2d), and treatment with NaOH to destroy the RNAs blocked the effect on *IL-1β* upregulation (Fig. 2e), suggesting that RNAs in L-MPs mediate IL-1β upregulation. Since TLR3 binds double-stranded RNA, TLR7 and TLR8 recognize single-stranded RNA and TLR9 senses unmethylated CpG DNA,^30–32^ we knocked down TLR3, TLR7, TLR8 or TLR9 in macrophages, which were then incubated with the L-MPs. We found that the knockdown of TLR3 but not TLR7, TLR8 or TLR9 inhibited L-MP-induced *IL-1β* expression (Fig. 2f, Supplementary Fig. 6), suggesting that L-MP-induced *IL-1β* expression is mediated via TLR3 signaling. NF-κB and MAPK are two key downstream molecules for TLR3 signaling. As expected, the activation of IκB (phosphorylated form) and MAPKs (phosphorylated ERK, p38 and JNK) was confirmed by western blot analyses (Fig. 2g). Consistently, inhibitors against NF-κB (BAY 11-7085), JNK (SP600125) or ERK (U0126) substantially inhibited L-MP-induced *IL-1β* expression (Fig. 2h). However, the p38 inhibitor SB203580 had no effect on the above *IL-1β* expression (Fig. 2h). These results suggest that TLR3 activation is required for IL-1β induction in macrophages by L-MPs.

### Noncoding RNAs enriched in L-MPs are potential ligands for TLR3

**Fig. 3:**
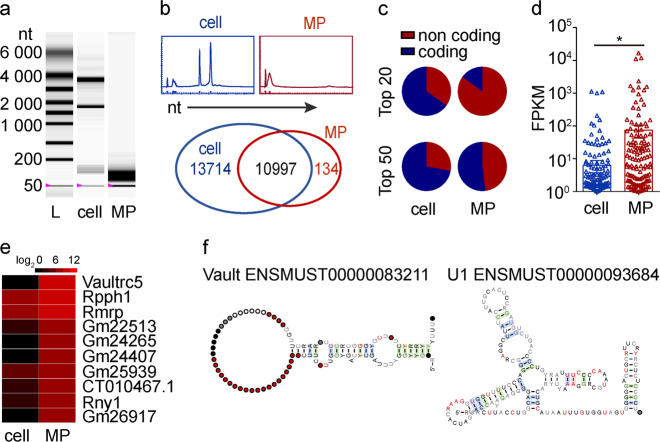
Noncoding RNAs enriched in L-MPs are potential ligands for TLR3. **a** RNAs isolated from Lewis cells or Lewis-MPs were extracted, and then, the RNA samples were separated by Experion RNA StdSens and HighSens Analysis Kits (Bio-Rad, Hercules, CA, USA). **b** The relative abundance of RNAs isolated from Lewis cells or Lewis-MPs was analyzed by Experion RNA StdSens and HighSens Analysis Kits (BIO-RAD) (upper). The RNAs were comprehensively sequenced using the HiSeq2500 system (Illumina, San Diego, CA, USA), and the sequences of 2 sets of RNA samples were compared (bottom). **c** Distribution of the coding and noncoding gene transcripts from the top enriched RNAs in Lewis cells or Lewis-MPs was analyzed. **d** The FPKM values of all noncoding RNAs in Lewis cells or Lewis-MPs were analyzed. **e** A heatmap of the top ten noncoding RNAs from Lewis-MPs was generated, and Lewis cells were used as a control. **f** The secondary structures of Vault misc RNA and U1 snRNA were downloaded from http://asia.ensembl.org/

Next, we investigated which RNAs in L-MPs mediated the activation of TLR3. Notably, RNAs isolated from parental tumor cells could not induce *IL-1β* expression (Fig. 2d), suggesting that unique RNA(s) in L-MPs is responsible for the TLR3 activation. An analysis of RNA distribution showed that the majority of RNA fragments in L-MPs (Lewis) were less than 200 nt; however, most RNA fragments from parental tumor cells were approximately 1800~3800 nt in length (Fig. 3a, b upper). We then conducted a comprehensive sequencing analysis (HiSeq2500) of RNAs isolated from L-MPs or parental lung tumor cells. We identified 11131 RNAs (coding and noncoding) in the L-MP group and 24711 RNAs in the parental tumor cell group. Among these RNAs, 10997 RNAs in the L-MP and parental tumor cell groups were identical (Fig. 3b bottom). Excluding these consistent RNAs, the top enriched RNAs in the L-MP group were noncoding RNAs. Specifically, nearly half of the top 50 and 17 of the top 20 enriched RNAs in the L-MP group were noncoding RNAs (Fig. 3c), and even the top 10 enriched RNAs were all noncoding RNAs, including misc RNA, small nuclear RNA, rRNA, ribozyme and lincRNA (Supplementary Fig. 7). In contrast, in the parental cell group, only 7 of the top 20 and 14 of the top 50 RNAs were noncoding RNAs (Fig. 3c). Furthermore, in all noncoding RNA analyses (fragments per kilobase of transcript per million fragments mapped, FPKM), we found that the noncoding RNAs in the L-MP group showed greater enrichment than those in the parental tumor cell group (Fig. 3d). In addition, the top ten enriched noncoding RNAs showed higher expression in the L-MP group than in the parental tumor cell group (Fig. 3e). Notably, most of the top ten enriched noncoding RNAs showed similar secondary structures with stem loops, including Vault misc RNAs, U1 snRNAs, U2 snRNAs and U4 snRNAs (Fig. 3f, Supplementary Fig. 8). Intriguingly, noncoding RNAs, especially snRNAs with stem-loop structures, can trigger TLR3 activation.^33,34^ These results, together with previous reports, suggest that highly enriched noncoding RNAs in L-MPs are potential ligands that activate TLR3 signaling in macrophages.

### L-MPs induce mitochondrial ROS to activate NLRP3 for IL-1β cleavage

**Fig. 4:**
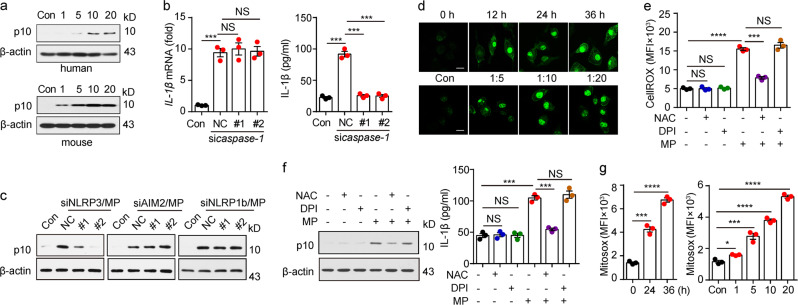
L-MP-induced mitochondrial ROS activate NLRP3 for IL-1β cleavage. **a** Human PBMC-derived macrophages (upper) or mouse BMDMs (bottom) were treated with HCC827-MPs or Lewis-MPs, respectively, at different doses. Then, the cells were collected, and the expression of active caspase-1 (p10) was detected by western blots. **b** Mouse BMDMs were transfected with or without caspase-1 siRNAs and then treated with Lewis-MPs. RNA and cultured medium were collected after 12 or 72 h of treatment, respectively. Then, IL-1β expression was analyzed by real-time PCR (left) and ELISAs (right). **c** BMDMs were transfected with or without NLRP3, NLRP1b or AIM2 siRNAs and then treated with Lewis-MPs for 24 h. Cells were collected, and the active caspase-1 levels were detected by western blots. **d** Mouse BMDMs were treated with Lewis-MPs at different times (upper) or doses (bottom). Then, the BMDMs were stained with CellROX and observed under a two-photon confocal microscope. Scale bar, 20 µm. **e**, **f** Mouse BMDMs were treated with Lewis-MPs in the presence or absence of NAC or DPI. The flt1 ROS fluorescence intensity of macrophages was measured via flow cytometry after 24 h. The active caspase-1 levels were detected by western blots (**f**, left) after 24 h, and the IL-1β expression was analyzed by ELISAs (**f**, right) after 72 h. **g** Mouse BMDMs were treated with Lewis-MPs at different times (left) and doses (right). Then, the BMDMs were stained with MitoSOX and measured by flow cytometry. Error bars indicate the mean ± SEM; *n* = 3 independent experiments. *P < 0.05, **P < 0.01, ***P < 0.001, ****P < 0.0001

Although activated TLR3 signaling can upregulate *IL-1β* mRNA expression and lead to the translation of pro-IL-1β, the production of mature IL-1β is mediated by inflammasome-activated caspase-1, which cleaves pro-IL-1β. When L-MPs (Lewis) were used to treat macrophages, we found that p10 (the active form of caspase-1) was strongly detected (Fig. 4a). However, although it did not affect the expression of *IL-1β* mRNA, knockdown of caspase-1 resulted in a strong decrease in the active form of IL-1β (Fig. 4b), suggesting that L-MPs induce IL-1β maturation via activating inflammasomes. Inflammasomes are commonly activated by oligomerization of NLRP3, NLRP1b or AIM2.^35^ Using siRNAs to knock down NOD-like receptors (NLRs) or AIM2, we found that NLRP3 siRNA, rather than other siRNAs, blocked IL-1β maturation induced by L-MPs (Fig. 4c), suggesting that L-MPs cause caspase-1 cleavage via activation of the NLRP3 inflammasome. Reactive oxygen species (ROS) are important factors that activate inflammasomes.^35,36^ Previously, we reported that melanoma-derived MPs increase ROS levels in DCs.^15^ Here, we also found that the ROS levels were elevated in L-MP-treated macrophages (Fig. 4d), and the ROS inhibitor N-acetylcysteine (NAC) reversed this change (Fig. 4e). L-MP-induced caspase-1 activation and IL-1β release were also abrogated by NAC (Fig. 4f). Consistent with these results, NAC treatment inhibited the binding of NLRP3 to the adapter protein ASC, which, however, had no effect on the expression of *caspase-1* and *IL-1β* mRNA (supplementary Fig. 9), suggesting that L-MPs activate NLRP3 via a ROS-dependent pathway. Given that L-MPs were taken up to lysosomes, where ROS can be generated through the NADPH oxidase NOX2 system in macrophages, we used the NOX2 inhibitor diphenylene iodonium (DPI) to block this pathway. However, DPI did not affect the ROS levels induced by L-MPs (Fig. 4e). In addition, DPI did not inhibit caspase-1 cleavage and IL-1β release, suggesting that L-MPs do not trigger the lysosomal pathway to generate ROS in macrophages (Fig. 4f). In addition to lysosomes, the mitochondrion is critical for ROS generation. Using MitoSOX, a specific mitochondrial ROS indicator, we found that the ROS levels were elevated in mitochondria after L-MP treatment (Fig. 4g), suggesting that L-MPs activate NLRP3 through a mitochondrial ROS-dependent pathway.

### Lysosomal calcium release induced by L-MPs causes mitochondrial ROS production

**Fig. 5:**
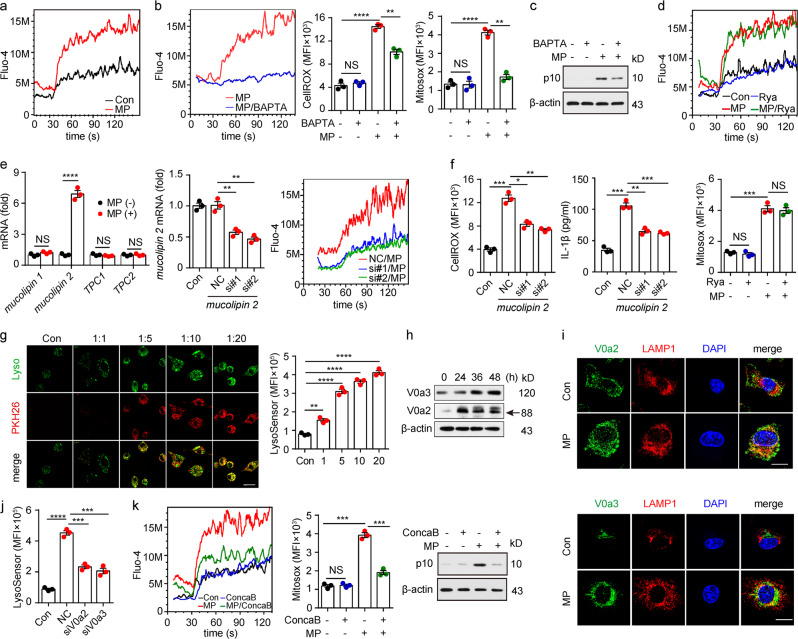
Lysosomal calcium release by L-MPs causes mitochondrial ROS production. **a** Mouse BMDMs treated with or without Lewis-MPs were loaded with the fluorescent Ca^2+^ indicator Fluo-4/AM. The Fluo-4 fluorescence intensity of the macrophages was measured via flow cytometry. Cells were stimulated with ionomycin (100 μM, 30 s). **b, c** Mouse BMDMs were treated with Lewis-MPs with or without BAPTA for 24 h, and then, the Ca^2+^ (**b**, left), ROS (**b**, middle), mitochondrial ROS (**b**, right) and active caspase-1 (**c**) levels of the macrophages were measured with flow cytometry or western blots. **d** Mouse BMDMs were treated with Lewis-MPs with or without ryanodine (Rya). After 24 h, the Ca^2+^ (*upper*) and mitochondrial ROS (*bottom*) levels of macrophages were measured via flow cytometry. **e**, **f** Mouse BMDMs were treated with or without Lewis-MPs. After 2 h, the cells were collected, and RNA was extracted for mRNA analysis of *mucolipin 1*, *mucolipin 2*, *TPC1* and *TPC2* by real-time PCR (**e**, left). The BMDMs were transfected with *mucolipin 2* siRNAs, and the silencing efficiency of the siRNAs was detected by real-time PCR (**e**, middle). The BMDMs transfected with *mucolipin 2* siRNAs were treated with Lewis-MPs. After 24 h, the Ca^2+^ (**e**, right) and ROS (**f**, left) levels of the macrophages were measured via flow cytometry. After 72 h, the culture medium was collected, and IL-1β expression was analyzed by ELISAs (**f**, right). **g** Mouse BMDMs were incubated with PKH26-labeled Lewis-MPs at different doses. After 12 h, the BMDMs were stained with LysoSensor. Then, the cells were observed under a two-photon confocal microscope. Scale bar, 20 µm (left). The flt1 LysoSensor fluorescence intensity of the macrophages was measured via flow cytometry (right). **h** Mouse BMDMs were treated with Lewis-MPs at different times. Then, the expression of V0a2 and V0a3 was detected via western blots. **i** Mouse BMDMs were treated with Lewis-MPs for 24 h. Immunofluorescence of V0a2 (green, upper), V0a3 (green, bottom) and LAMP1 (red) in the control and Lewis-MPs groups was assessed with two-photon confocal microscopy. Scale bar, 20 µm. **j** Mouse BMDMs were transfected with V0a2 or V0a3 siRNAs and then treated with Lewis-MPs. After 12 h, the cells were stained with LysoSensor, and the flt1 LysoSensor fluorescence intensity of the macrophages was measured by flow cytometry. **k** Mouse BMDMs were treated with Lewis-MPs with or without concanamycin B (ConcaB). After 24 h, the cells were stained with Fluo-4 (left) or MitoSox (middle). Then, the Fluo-4 and MitoSox fluorescence intensity of the macrophages was measured by flow cytometry. The active caspase-1 levels were detected by western blots (right). Error bars indicate the mean ± SEM; *n* = 3 independent experiments. *P < 0.05, **P < 0.01, ***P < 0.001, ****P < 0.0001

Next, we investigated the molecular pathway through which L-MPs (Lewis) triggered ROS production in mitochondria. Ca^2+^ overload can induce mitochondria to overproduce ROS.^37^ Intriguingly, cytosolic Ca^2+^ levels in macrophages substantially increased upon L-MP treatment (Fig. 5a), and the Ca^2+^ antagonist BAPTA could decrease the ROS levels and inhibit pro-caspase-1 cleavage (Fig. 5b, c), suggesting that L-MPs mobilize Ca^2+^ to promote mitochondrial ROS production. Then, we explored how L-MPs induced the increase in cytosolic Ca^2+^. The endoplasmic reticulum is thought to be the organelle that stores Ca^2+^. However, blocking ER Ca^2+^ release with an inhibitor did not alter the levels of cytosolic Ca^2+^ and mitochondrial ROS in L-MP-treated macrophages (Fig. 5d). In addition to the ER, lysosomes also store and release Ca^2+^ through Ca^2+^ transporters, such as two pore channels (TPCs) and mucolipin transient receptor potential channels (TRPMLs).^38,39^ Among these transporters, *mucolipin 2* (TRPML2) was upregulated upon L-MP treatment, and blocking *mucolipin 2* decreased the cytosolic Ca^2+^ level, concomitant with a decrease in mitochondrial ROS as well as IL-1β maturation (Fig. 5e, f). These results indicated that L-MPs indeed trigger lysosomal Ca^2+^ release to mediate ROS production. We then further investigated how L-MPs triggered the release of lysosomal Ca^2+^. Lysosomal homeostasis requires a stable acidic microenvironment, which is maintained by V-ATPase that pumps H^+^ from the cytosol to the lysosomal lumen.^40^ Intriguingly, L-MP treatment resulted in a decrease in lysosomal pH (Fig. 5g). This change was supported by the increase in the V-ATPase subunits V0a2 and V0a3 on lysosomal membranes, as evidenced by western blot and immunofluorescence analyses (Fig. 5h, i). However, knocking down V0a2 or V0a3 via siRNAs could attenuate the effect of L-MPs on lysosomal pH, suggesting that L-MPs may promote lysosome acidification through regulating V-ATPase (Fig. 5j). To further verify the role of lysosomal pH in L-MP-triggered lysosomal Ca^2+^ release, we used concanamycin B, a V-ATPase-specific inhibitor, to increase the lysosomal pH. As a result, the cytosolic Ca^2+^ and mitochondrial ROS levels were reduced in L-MP-treated macrophages, concomitant with attenuated caspase-1 cleavage (Fig. 5k). Together, these data suggested that L-MPs can trigger the release of lysosomal Ca^2+^ into the cytosol via decreasing the lysosomal pH, which promotes mitochondrial ROS production and the subsequent NLRP3 inflammasome activation.

### L-MP-induced IL-1β produced by macrophages promotes lung tumor development

**Fig. 6:**
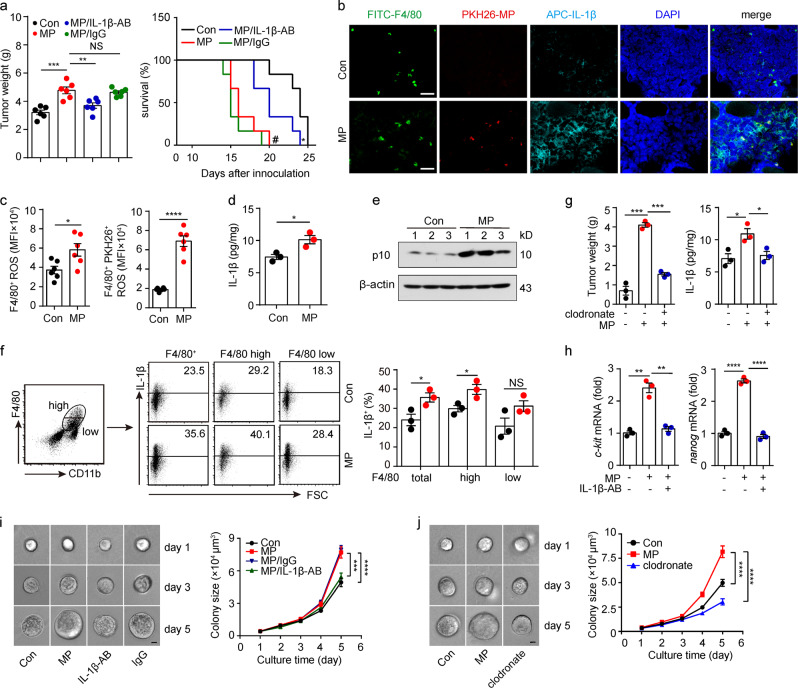
L-MP-induced IL-1β generated by macrophages promotes lung tumor development. **a–g** First, 5 × 10^4^ Lewis tumor cells were injected into the right thigh muscle of the mice. After 10 days, 5 × 10^6^ Lewis-MPs were injected into the tumor once every 2 days for a total of three times. Two groups of mice (*n* = 12) were treated with purified IL-1β antibody (IL-1β-AB, 50 µg) or IgG (50 µg) on days 9 and 12, respectively. On day 15, half of the mice (*n* = 6) in each group were sacrificed, and the tumor weight was measured (left). The remaining mice (*n* = 6) were used for the long-term survival observation (right). ^#^*P* < 0.001, MP group compared with Con group. *P < 0.05, MP group compared with MP/IL-1β-AB group. **b** Tumor sections were labeled with immunofluorescence to indicate the distribution of the macrophages (F4/80, green) and IL-1β (navy blue). The distribution of Lewis-MPs (red) was also recorded. Cell nuclei were stained with DAPI (blue). Scale bar, 30 µm. **c** Leukocytes in the above tumor tissues were isolated, and then, the ROS levels of tumor-infiltrating macrophages (CD11b^+^ F4/80^+^) were measured by flow cytometry (*n* = 6). **d** The IL-1β expression of tumor tissues was measured by using an ELISA kit. **e** The active caspase-1 levels of tumor tissues were measured via western blots. **f** The IL-1β expression of tumor-infiltrating macrophages (CD11b^+^ F4/80^+^) was measured by flow cytometry. **g** Clodronate (FormuMax Scientific Inc., CA) was i.p. injected into the mice on day 9 (200 µl) and day 12 (100 µl) after tumor inoculation. On day 15, the mice were sacrificed, and the tumor weight was measured (left). The IL-1β expression of the tumor tissues was measured by using an ELISA kit (right). **h** *c-kit* and *nanog* mRNA expression of the tumor tissues from mice in **a** was measured by real-time PCR. **i** The isolated tumor cells from mice in **a** were seeded in soft 3D fibrin gels. The tumor colony (n = 150) size was analyzed. Scale bar, 20 µm. **j** The isolated tumor cells from mice in **g** were seeded in soft 3D fibrin gels. The tumor colony (*n* = 150) size was analyzed. Scale bar, 20 µm. Error bars indicate the mean ± SEM; *n* = 3 independent experiments unless otherwise indicated. *P < 0.05, **P < 0.01, ***P < 0.001, ****P < 0.0001

Next, we investigated the role of IL-1β produced from L-MP-treated macrophages in lung tumors. Using the Lewis lung cancer model, we found that the injection of L-MPs (Lewis) into the tumors resulted in accelerated tumor growth in the muscle and shortened the survival time of the mice, but these changes were blocked by an IL-1β neutralizing antibody (Fig. 6a). Consistently, abundant IL-1β was found in the lung tumor tissue and could be further augmented by L-MP injection (Fig. 6b). As a result, the intensity of the CD31^+^ microvessels was enhanced at the tumor site (supplementary Fig. 10); the number of apoptotic tumor cells was decreased, as evidenced by TUNEL assays, and tumor cell proliferation was augmented (Supplementary Fig. 11). However, the injection of L-MPs did not appear to significantly affect immune cells. We found that L-MP injection only slightly elevated the proportion of macrophages and CD8^+^ T cells in the tumor tissues, which did not alter the immune cell proportions in the spleen, lymph nodes, or peripheral blood (Supplementary Fig. 12). Following injection, the L-MPs were found to be effectively taken up by tumor-infiltrating macrophages (Fig. 6b). Consistent with the in vitro data, these tumor-infiltrating macrophages also showed increased ROS levels, enhanced caspase-1 activity and elevated mature IL-1β production following L-MP treatment (Fig. 6c–f). However, when we used chlodronate liposomes to deplete the macrophages, the L-MP-mediated promotion of IL-1β production and tumor growth was blocked (Fig. 6g), suggesting that IL-1β produced by L-MP-treated macrophages promotes lung tumor development. The tumor-promoting effect of IL-1β might be mediated through multiple mechanisms. In addition to its tumor angiogenesis-promoting effect,^41^ IL-1β has also been reported to promote tumor cell stemness, a fundamental biological event that is critical for tumorigenesis.^42^ We thus analyzed stemness-related genes in CD45^−^ tumor cells and found that *c-kit* and *nanog* were upregulated in the L-MP group, but this change was blocked by the neutralization of IL-1β (Fig. 6h). These results suggested that L-MP-induced IL-1β is involved in tumor cell stemness. Despite the importance of stem cell-like tumor cells in tumor initiation, progression, metastasis and drug resistance, this population belongs to a minor subpopulation, and the insufficient number of cells restricts extensive mechanistic studies on stem cell-like tumor cells. To overcome this limitation, we previously established a mechanics-based 3D soft fibrin gel culture system to select and amplify highly tumorigenic melanoma-repopulating cells (TRCs).^25,43^ Here, we further determined the effect of IL-1β on TRCs in lung tumor tissues. After the L-MP injection, the isolated tumor cells were seeded into the soft 3D fibrin gels. L-MP treatment effectively promoted TRC growth in the gels (Fig. 6i); however, this effect was blocked by IL-1β neutralization (Fig. 6i). The L-MP-mediated promotion of TRCs was also inhibited by chlodronate liposome-mediated macrophage depletion (Fig. 6j). In addition, we performed an in vitro experiment using IL-1β to treat fibrin gel-cultured TRCs. We found that IL-1β treatment upregulated *c-kit* and *nanog* expression and increased the TRC colony size (Supplementary Fig. 13). Together, these data suggest that L-MP-induced IL-1β produced by macrophages promotes lung tumor development.

### L-MPs facilitate tumor growth in a humanized mouse model

**Fig. 7:**
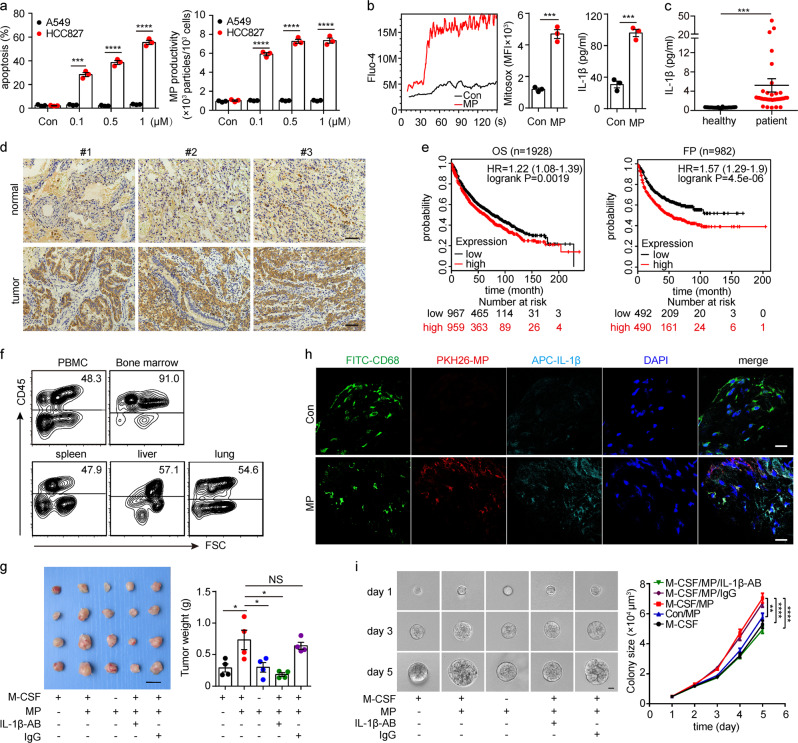
hL-MPs facilitate tumor growth in a humanized mouse model. **a** Human HCC827 lung cancer cells were treated with different concentrations of the mutated EGFR inhibitor icotinib. After 24 h, the cell viability was analyzed with PI and annexin V staining, and the number of HCC827-MPs was calculated by flow cytometry. **b** Human PBMC-derived macrophages were treated with HCC827-MPs. After 24 h, the Ca^2+^ (left) and mitochondrial ROS (middle) levels of the macrophages were measured by flow cytometry. After 72 h, the culture medium was collected, and IL-1β expression was analyzed by ELISAs (right). **c** Lung cancer patient (*n* = 34) and healthy human (*n* = 38) peripheral blood samples were collected. Then, the IL-1β serum concentrations were analyzed by ELISAs. **d** Immunohistochemical staining of IL-1β in patients’ lung cancer tissues and paracancerous normal lung tissues was analyzed. Scale bar, 100 µm. **e** The association between IL-1β and the survival of patients with lung cancer was analyzed based on a Kaplan-Meier plot (http://kmplot.com/analysis/). **f** A humanized mouse model (*n* = 4) was established as described in the “Methods” section. Nine weeks after CD34^+^ HSC transplantation, PBMCs, bone marrow cells, and lymphocytes from the spleen, liver and lung were stained with a human CD45 antibody and analyzed by flow cytometry. **g** First, 5 × 10^6^ HCC827 tumor cells were injected into the right femur muscle of 10-week-old humanized mice (*n* = 4). Two groups of mice were treated with neutralizing IL-1β-AB (50 µg) or IgG (50 µg) at 8 weeks after HSC transplantation twice weekly. The day after the antibody injection, the mice were injected with 5 × 10^6^ HCC827-MPs into the tumor once every 2 days. Twenty three days after the tumor inoculation, the mice were sacrificed, and the tumor weight was measured. Scale bar, 20 mm. **h** Tumor sections were labeled via immunofluorescence assays to indicate the distribution of the macrophages (CD68^+^, green) and IL-1β (navy blue). Cell nuclei were stained with DAPI. Scale bar, 20 µm. **i** Human tumor cells isolated from tumor-bearing humanized mice were seeded in a 3D culture system, and the colony (*n* = 150) size was analyzed. Scale bar, 20 µm. Error bars indicate the mean ± SEM; *n* = 3 independent experiments unless otherwise indicated. *P < 0.05, **P < 0.01, ***P < 0.001, ****P < 0.0001

Next, we investigated whether L-MP-induced IL-1β produced by macrophages could be utilized for human lung cancer. Given the frequency of EGFR mutations in lung cancer patients, we treated EGFR-mutated (E746-A750 deletion) HCC827 human lung cancer cells with the mutated EGFR inhibitor icotinib. This treatment led to the release of abundant hL-MPs (Fig. 7a). These hL-MPs consistently induced lysosomal Ca^2+^ release, increased mitochondrial ROS levels and promoted IL-1β release in the treated macrophages (Fig. 7b). Consistent with this in vitro result, IL-1β levels increased in lung cancer patients compared to the healthy controls (Fig. 7c). The immunohistochemical staining analysis also showed that IL-1β was present in patients’ lung cancer tissues (Fig. 7d). Consistent with these results, the Kaplan–Meier plot (http://kmplot.com/analysis/) analysis showed that the IL-1β levels were correlated with poor survival of patients with lung cancer (Fig. 7e). To further validate the role of L-MPs in lung cancer patients, we used a humanized mouse model.^26^ After 9 weeks of human hematopoietic stem cell (HSC) transplantation, we identified humanized mice in which 90% of the bone marrow cells were positive for human CD45 (Fig. 7f, Supplementary Fig. 14). The mice were also injected with a human M-CSF-expressing plasmid to develop humanized macrophages, as shown by human CD68 immunostaining (supplementary Fig. 15). Next, these humanized mice were intramuscularly injected with HCC827 cells to induce lung tumors. As expected, the injection of HCC827-derived L-MPs facilitated tumor growth, concomitant with increased IL-1β levels in tumor tissue (Fig. 7g, h). This enhanced tumor growth, however, was blocked by IL-1β neutralization or macrophage depletion (Fig. 7g). However, HCC827 cells isolated from the above humanized mice were seeded in soft 3D fibrin gels. Consistently, TRCs in the hL-MPs group grew much better than those in the control group, and this phenomenon was also dependent on IL-1β and macrophages (Fig. 7i). Together, these results suggest that hL-MP-affected macrophages promote human lung cancer development via secretion of IL-1β.

## Discussion

Chronic inflammation is commonly found with the initiation, promotion, and progression of tumorigenesis.^1,44^ However, tumor microenvironments are highly immunosuppressive.^45,46^ To date, our knowledge of how tumor inflammation and immunosuppression are orchestrated by macrophages, the most abundant innate immune cells in the tumor microenvironment, is still incomplete; this process promotes tumor development. In this study, we found that human lung cancer cell-released MPs trigger the TLR3 and NLRP3 inflammasome pathways in macrophages, leading to the secretion of IL-1β. Thus, macrophages exposed to tumors may become inflammatory M2 tumor-associated macrophages to promote human lung cancer development.

MPs integrate various biological information. Although tumor MPs are derived from tumor cells, their components are actually different from the parent cells. First, the membrane structures are different because MP membranes but not cellular membranes are resistant to the detergent Triton X-100, indicating that lipid rafts might be the main membrane component of MPs.^47^ Second, the packaged contents are different. Although tumor MPs contain enzymes, lipids, RNAs and even DNA, these biomolecules seem to be functionally different from their counterparts in the parent cells. For instance, DNA components from tumor MPs rather than parental tumor cells can activate the cGAS-STING pathway.^14^ In this study, we further found that lung cancer MPs contain noncoding RNAs that can activate TLR3; however, noncoding RNAs from parental tumor cells or normal cells do not have this ability, indicating that the spectrum of RNAs was altered during MP formation. Thus, our present findings further indicate the uniqueness of tumor MPs as a source of communication. Macrophages are a major source of IL-1β production following various extracellular signals. Although the TLR3 signaling pathway activation leads to the upregulation of *IL-1β* expression and the generation of pro-IL-1β, the latter needs to be further processed into its mature form via the inflammasome pathway.

Inflammasomes are multiprotein signaling platforms that trigger the cleavage of pro-caspase-1 into active caspase-1, and the latter then cleaves pro-IL-1β and pro-IL-18 into their active forms (IL-1β and IL-18).^48^ A majority of inflammasomes are formed by NLRs^49^. Using siRNA technology, we found that NLRP3 mediates L-MP-activated inflammasomes. However, we wondered how L-MPs activate the NLRP3 inflammasome. Several pathways that activate NLRP3 have been identified, including K^+^ efflux, mitochondrion-derived ROS, released mitochondrial DNA, and released lysosomal cathepsins.^50–52^ We found that ROS levels in mitochondria were elevated after L-MP treatment, which contributes to the L-MP-activated inflammasome. We further demonstrated that after phagocytosis by macrophages, L-MPs in lysosomes activate lysosomal Ca^2+^ channels, leading to Ca^2+^ release to the cytosol. As a result, cytosolic Ca^2+^ increases mitochondrial ROS. Although this mechanism was elucidated, a key issue is how L-MPs trigger lysosomal Ca^2+^ release, which is important to understand the biology of tumor MPs. The lysosome is a key organelle, not only for degrading materials but also for storing Ca^2+^. This Ca^2+^ can be released into the cytoplasm via calcium ion channels on lysosomal membranes. Following entry into macrophages, L-MPs are found in the lysosomes and activate the calcium channel TRPML2, resulting in lysosomal Ca^2+^ release probably through decreased lysosomal pH. In turn, this released Ca^2+^ triggers mitochondrial ROS production to activate the NLRP3 inflammasome for mature IL-1β release. Previous studies have observed that exosomes induce IL-1β production in macrophages,^53–55^ but the mechanism is unclear. Although exosomes can also be taken up by macrophages via phagocytosis, they might not use the same mechanism as L-MPs use to produce IL-1β, given the differences between exosomes and MPs in membrane components, size and contents. Despite this, how exosomes regulate IL-1β production in macrophages should be determined to further elucidate the nature of exosomes and MPs. Recently, studies have shown that UV-irradiated apoptotic cancer cells inhibit cancer progression and lung metastasis through exosome-mediated macrophage reprogramming,^56^ suggesting multiple functions of tumor cell-derived EVs in the tumor microenvironment.

In summary, our data showed that human lung cancer cell-derived MPs, due to their ability to carry unique noncoding RNAs, result in the activation of TLR3 and the subsequent pro-IL-1β production in macrophages. Meanwhile, these L-MPs trigger lysosomal Ca^2+^ release through the activated calcium channel TRPML2 by decreasing the pH. This released Ca^2+^ promotes mitochondrial ROS production, thus activating the inflammasome for mature IL-1β production. This release of IL-1β by macrophages exerts a tumor-promoting effect, which may help explain target therapy-related tumor progression in EGFR mutant lung cancer patients.

## Supplementary information

supplementary information
