# Supplementary material for: Macrophages reprogrammed by lung cancer microparticles promote tumor development via release of IL-1β
Source: Cell Mol Immunol. 2019 Oct 24;17(12):1233–44. doi: 10.1038/s41423-019-0313-2 (PMC7784894; doi:10.1038/s41423-019-0313-2)
Supplement: Supplementary file 1 — supplementary information [file 41423_2019_313_MOESM1_ESM.pdf]

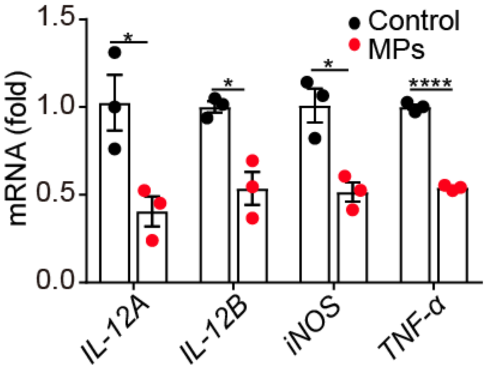

Supplementary Fig. 1 Related to Fig. 1

Human PBMCs derived macrophages were treated with HCC827-MPs at the ratio 1: 20 (macrophages: MPs). 12 h later, *IL-12*, *iNOS* and *TNF-α* expression was analyzed by real-time PCR. Error bars indicate mean ± SEM; n=3 independent experiments. *$P < 0.05$, ****$P < 0.0001$.

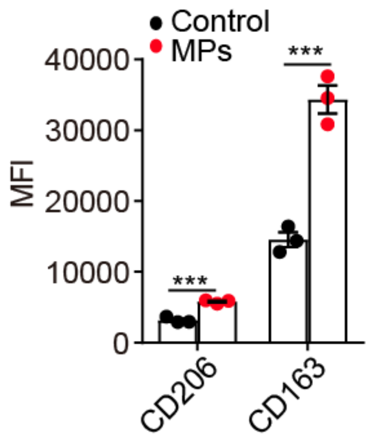

Supplementary Fig. 2 Related to Fig. 1

Human PBMCs derived macrophages were treated with HCC827-MPs at the ratio 1: 20 (macrophages: MPs). 24 h later, mean fluorescence intensity of CD163 and CD206 expression was analyzed by flow cytometry. Error bars indicate mean ± SEM; n=3 independent experiments. ***$P < 0.001$.

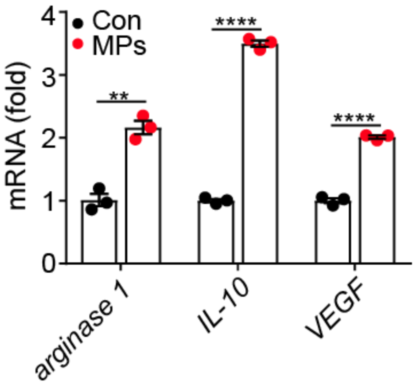

Supplementary Fig. 3 Related to Fig. 1

Human PBMCs derived macrophages were treated with 100ng/ml LPS for 12 h and then incubated with HCC827-MPs at the ratio 1: 20 (macrophages: MPs). Another 12 h later, *IL-10*, *VEGF* and *arginase-1* expression were analyzed by real-time PCR. Error bars indicate mean ± SEM; n=3 independent experiments. **$P < 0.01$, ****$P < 0.0001$.

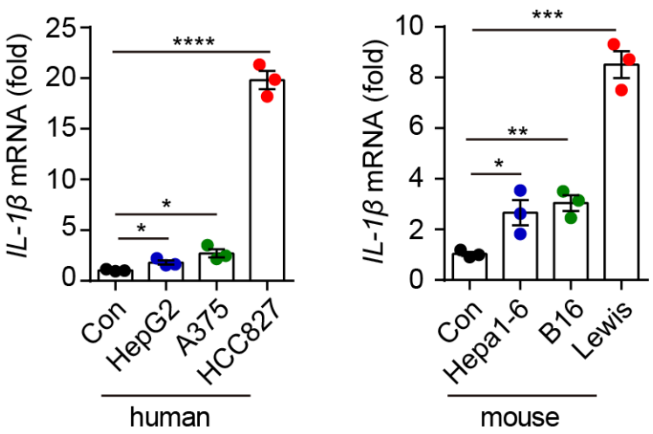

Supplementary Fig. 4 Related to Fig. 1

Human PBMCs derived macrophages (left) were treated with HepG2-MPs, A375-MPs or HCC827-MPs at the ratio 1: 20 (macrophages: MPs). 12 h later, *IL-1β* expression was analyzed by real-time PCR. Mouse BMDMs (right) were treated with Hepa1-6-MPs, B16-MPs or Lewis-MPs at the ratio 1: 20 (macrophages: MPs) for 12 h. Then *IL-1β* mRNA levels were analyzed by real-time PCR. Error bars indicate mean ± SEM; n=3 independent experiments. *$P < 0.05$, **$P < 0.01$, ***$P < 0.001$, ****$P < 0.0001$.

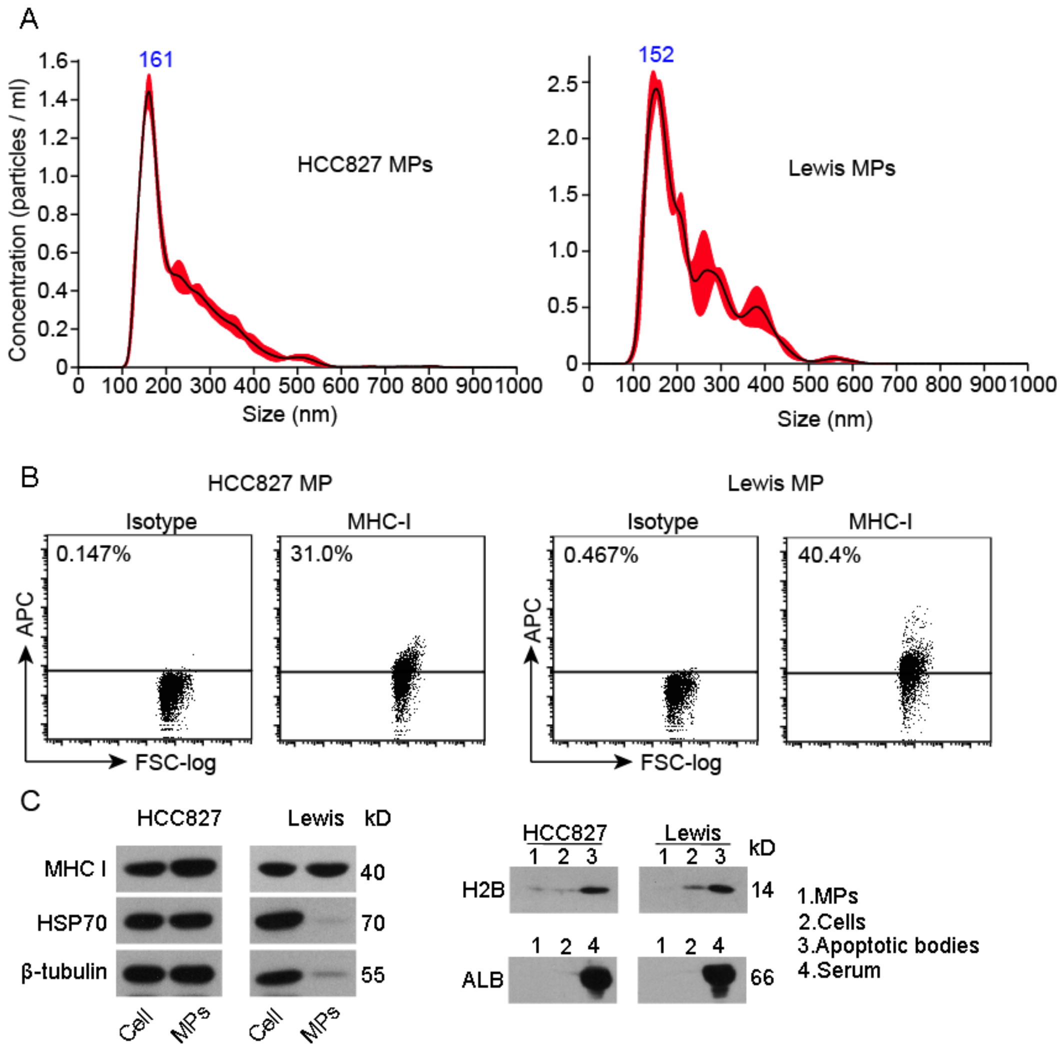

Supplementary Fig. 5 Related to Fig. 1

The sizes of HCC827-MPs and Lewis-MPs were detected by Nanoparticle tracking analysis (NS300, Malvern) (A); MHC class I on the surface of HCC827-MPs and Lewis-MPs were detected by flow cytometry (B); MHC class I, Heat shock protein 70 (HSP 70), β-tubulin, histone H2B and apolipoprotein B(ALB) of MPs were detected by western blotting, cells, apoptotic bodies and serum were as positive or negative controls (C).

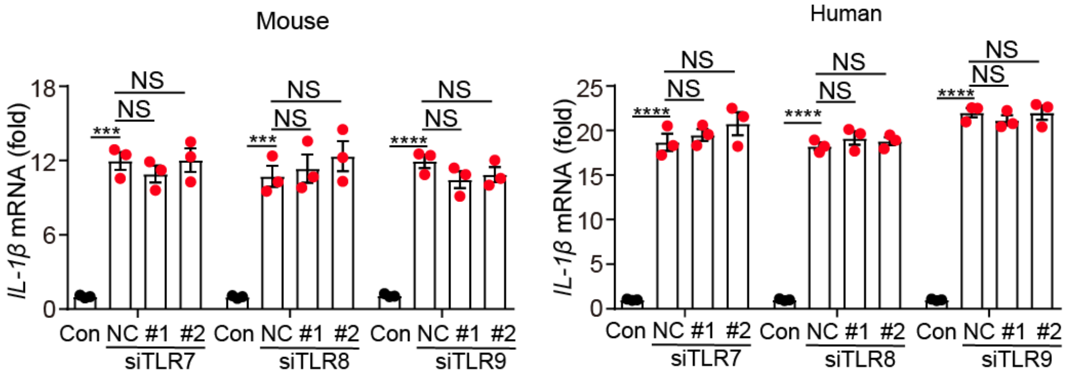

Supplementary Fig. 6 Related to Fig. 2

Mouse BMDMs (left) or human PBMCs derived macrophages (right) were transfected with *TLR7*, *TLR8* or *TLR9* siRNA, and then treated with Lewis-MPs or HCC827-MPs respectively. 12 h later, *IL-1β* mRNA levels were analyzed by real-time PCR. Error bars indicate mean ± SEM; n=3 independent experiments. ***$P < 0.001$, ****$P < 0.0001$.

Top enriched NO.	Gene short name	Ensembl ID	Type
1	Vaultrc5	ENSMUSG00000065145.1	misc RNA
2	Rpph1	ENSMUSG00000092837.1	ribozyme
3	Rmrp	ENSMUSG00000088088.1	ribozyme
4	Gm22513	ENSMUSG00000096349.1	snRNA
5	Gm24265	ENSMUSG00000096243.1	snRNA
6	Gm24407	ENSMUSG00000094377.1	snRNA
7	Gm25939	ENSMUSG00000093843.1	snRNA
8	CT010467.1	ENSMUSG00000106106.2	rRNA
9	Rny1	ENSMUSG00000065701.1	misc RNA
10	Gm26917	ENSMUSG00000097971.3	lincRNA

Supplementary Fig. 7 Related to Fig. 3

Ensembl ID and type of the top 10 enriched non-coding RNAs in L-MPs were shown and available from the database http://asia.ensembl.org.

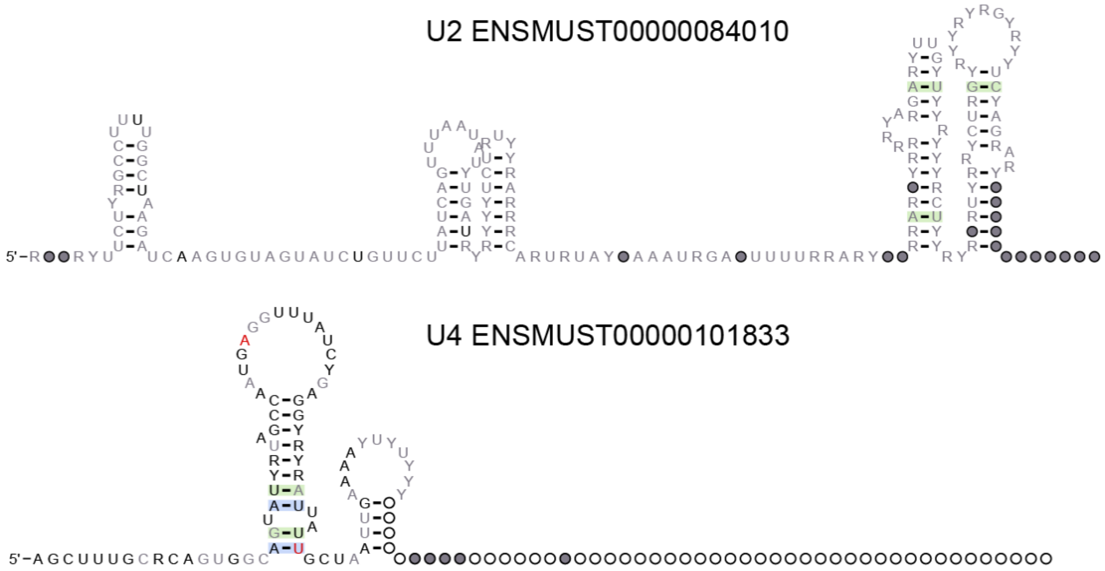

Supplementary Fig. 8 Related to Fig. 3

The secondary structure of U2 and U4 was shown and available from the database http://asia.ensembl.org

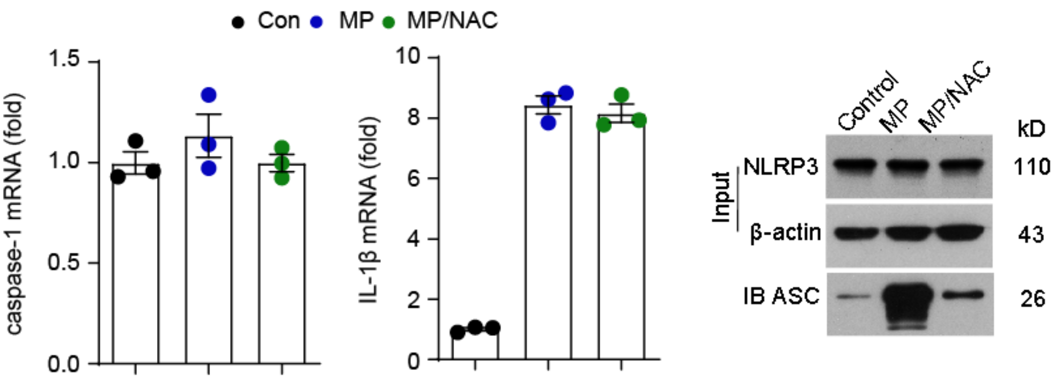

Supplementary Fig. 9 Related to Fig. 4

Mouse BMDMs were treated with Lewis-MPs at the ratio 1: 20 (macrophages: MPs) for 24h in the presence of NAC or not. Cells were collected, *caspase-1* and *IL-1β* mRNA levels were analyzed by real-time PCR (A); Cell lysate were pulled down by anti-NLRP3 antibody and then ASC were detected by western blotting (B).

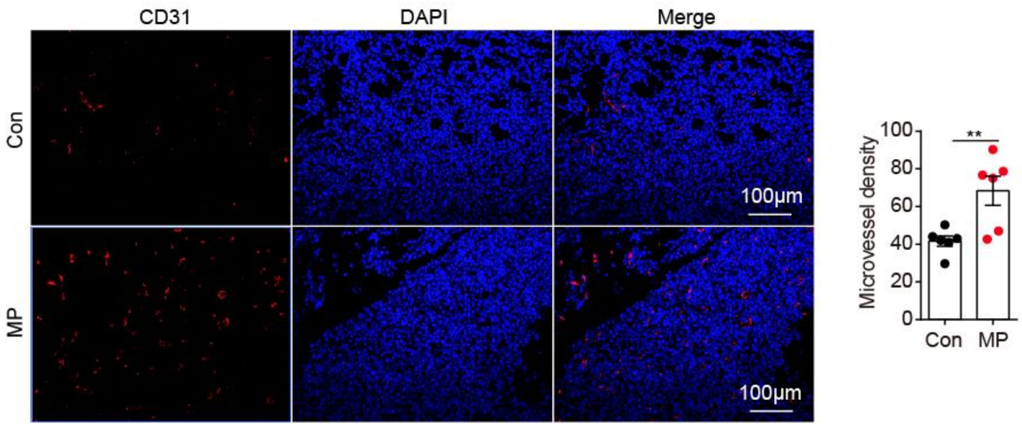

Supplementary Fig. 10 Related to Fig. 6

5×10^4 Lewis tumor cells were injected into the right thigh muscle of mice. Ten days later, 5×10^6 Lewis-MPs were injected into tumor once two days for three times. On day 15, mice were sacrificed and tumor tissue sections were stained with CD31 antibody and the density were counted by random visual field. Error bars indicate mean ± SEM. ***P* < 0.01. Scale bar: 100 μm.

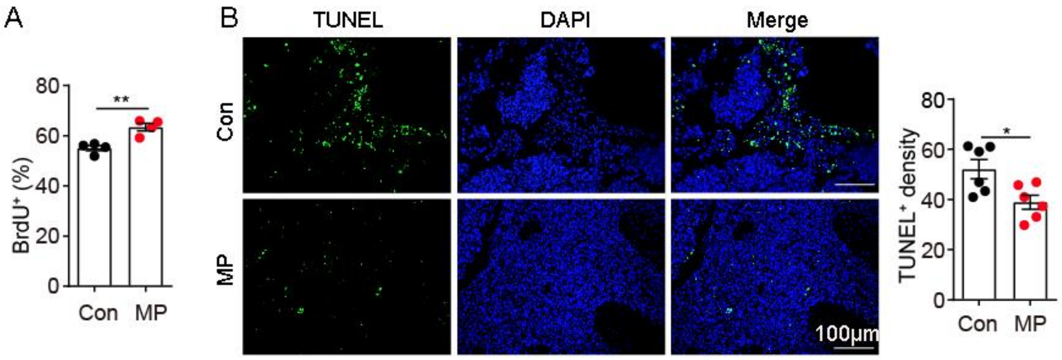

Supplementary Fig. 11 Related to Fig. 6

5×10^4 Lewis tumor cells were injected into the right thigh muscle of mice. Ten days later, 5×10^6 Lewis-MPs were injected into tumor once two days for three times. On day 15, mice were sacrificed, tumor cells were collected and incubated with BrdU for 1h and then cells were stained with FITC labeled anti-BrdU antibody and analyzed by flow cytometry (A); tumor tissue section were analyzed by TUNEL assay and TUNEL positive cells were counted by fluorescence microscope (B). Error bars indicate mean ± SEM. *P < 0.05, **P < 0.01. Scale bar: 100 μm.

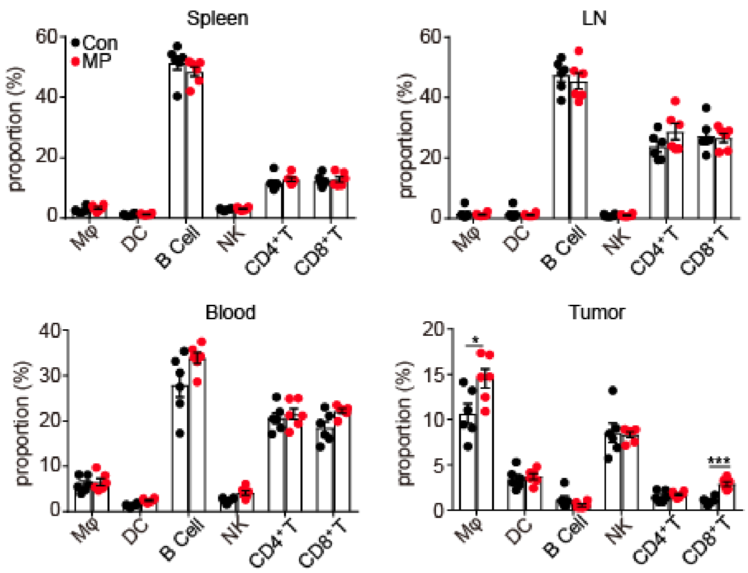

Supplementary Fig. 12 Related to Fig. 6

5×10^4 Lewis tumor cells were injected into the right thigh muscle of mice. Ten days later, 5×10^6 Lewis-MPs were injected into tumor once two days for three times. On day 15, mice were sacrificed, and immune cells proportion in the spleen, lymph node (LN), peripheral blood and tumor were analyzed by flow cytometry. Error bars indicate mean ± SEM. *P < 0.05, ***P < 0.001.

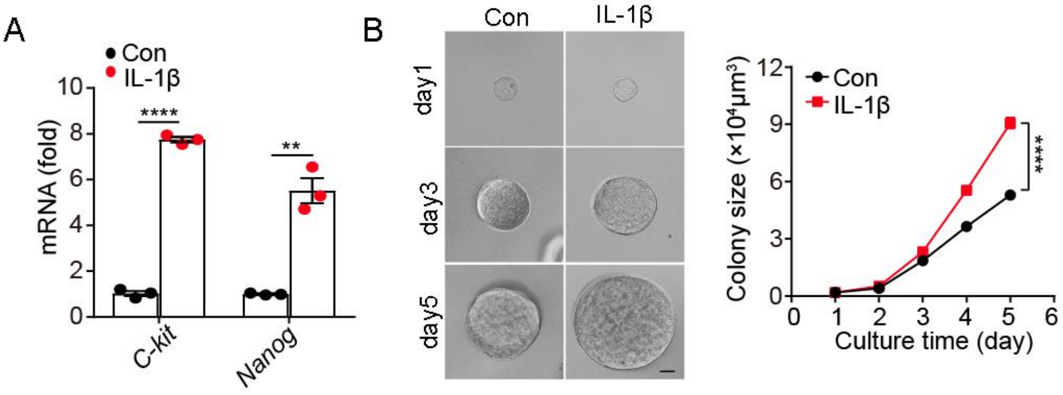

Supplementary Fig. 13 Related to Fig. 6

Lewis cells were seeded in 90 pa 3D fibrin gels in the presence of 1ng/ml IL-1β or not. TRCs were collected on day 5, and then *c-kit* and *nanog* mRNA were detected by real time PCR(A); The tumor colony size was observed and analyzed under microscope (B). Error bars indicate mean ± SEM. $^{**}P < 0.01$, $^{****}P < 0.0001$. Scale bar: 20 μm.

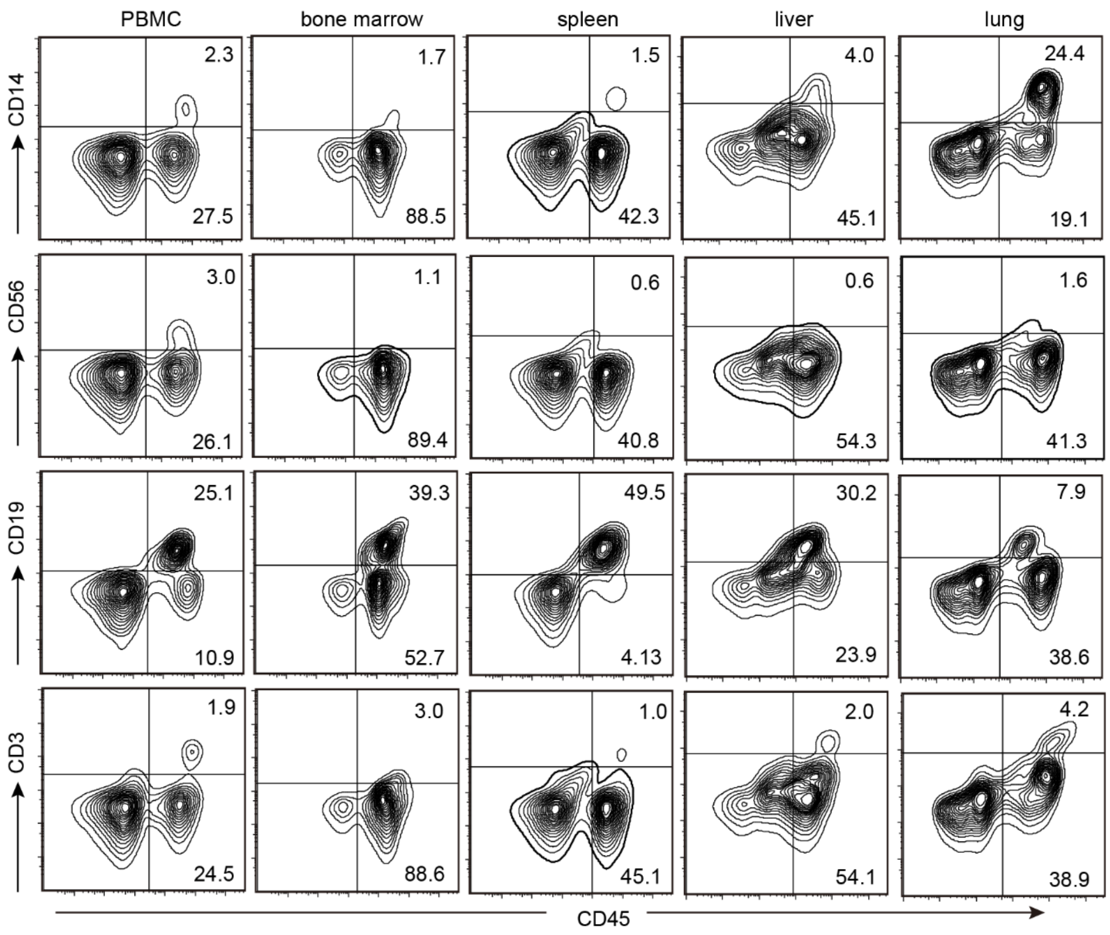

Supplementary Fig. 14 Related to Fig. 7

Humanized mice model was established as described in methods. 9 weeks after $CD34^+$ HSCs transplantation, cells from PBMCs, bone marrow, and lymphocytes from spleen, liver and lung were stained with human CD45, CD3, CD19, CD56 or CD14 antibody and analyzed by flow cytometry.

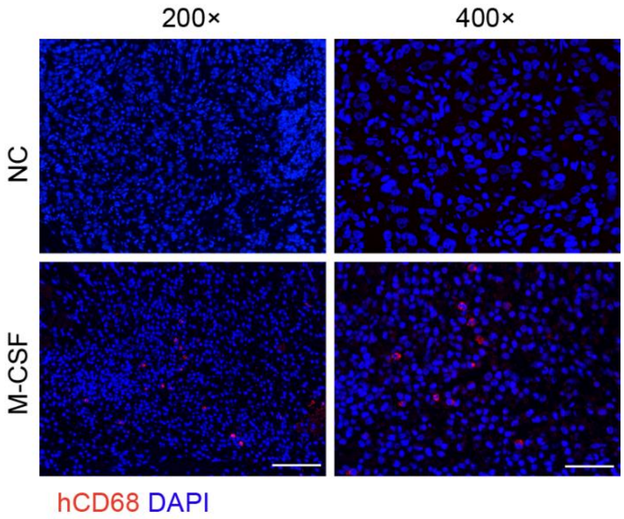

Supplementary Fig. 15 Related to Fig 7

Tumor sections were labeled by immunofluorescence assay to indicate the distribution of human macrophages (CD68 red). Cell nucleus was stained by DAPI (blue). Scale bar for 200×, 100 μm. Scale bar for 400×, 200 μm.

Supplementary Table 1

Gene name	Sequence	Gene name	Sequence
mouse			
si-caspase-1-1	GAAGGCCCATATAGAGAAA	si-NLRP3-1	GTACTTAAATCGTGAAACA
si-caspase-1-2	CCAAGGTGATCATTATTCA	si-NLRP3-2	CAGCCAGAGTGGAATGACA
si-*NLRP1b*-1	GCAAGGAATTCCAACTCTT	si-*TLR3*-1	GAATCTTACTCACAACCAA
si-*NLRP1b*-2	GCTGCCAACTAAAGACTTT	si-*TLR3*-2	GTATTGAACCTGCAACATA
si-*AIM2*-1	GGAACAGGCTGCTACAGAA	si-*TLR7*-1	CTAGAGCTCTATCTTTATA
si-*AIM2*-2	GGTCACCAGTTCCTCAGTT	si-*TLR7*-2	CAACAACCGGCTTGATTTA
si-*mucolipin 2*-1	GCTGTAGCATATACACTCA	si-*TLR8*-1	CCAATACTCAAGTGTTTAA
si-*mucolipin 2*-2	GCAGTTCATTCCCGAGAGA	si-*TLR8*-2	CTACCAAGTTCTCTAAGGA
si-*TLR9*-1	GCCTCTCCTTGATCTCCAA		
si-*TLR9*-2	CCATCTGTCTCTGAAGTAT		
human			
si-*TLR3*-1	GCACGAATTTGACTGAACT	si-*TLR7*-1	GGGTATCAGCGTCTAATAT
si-*TLR3*-2	GTTGAACCTTACCCATAAT	si-*TLR7*-2	AATTGCCCTCGTTGTTATA
si-*TLR8*-1	GAACGGAAATCCCGGTATA	si-*TLR9*-1	GCAGACACTGTGTGCACAT
si-*TLR8*-2	CTTCCAAACTTATCGACTA	si-*TLR9*-2	CCCACCAGCTAATCCTGTT

Supplementary Table 2

Gene name	Forward primer	Reverse primer
human		
β-actin	CATTGCTGACAGGATGCAGAAGG	TGCTGGAAGGTGGACAGTGAGG
IL-10	TCTCCGAGATGCCTTCAGCAGA	TCAGACAAGGCTTGGCAACCCA
VEGF	TTGCCTTGCTGCTCTACCTCCA	GATGGCAGTAGCTGCGCTGATA
arginase 1	TCATCTGGGTGGATGCTCACAC	GAGAATCCTGGCACATCGGGAA
IL-1β	TGGACCTTCCAGGATGAGGACA	GTTCATCTCGGAGCCTGTAGTG
mouse		
β-actin	CATTGCTGACAGGATGCAGAAGG	TGCTGGAAGGTGGACAGTGAGG
IL-1β	TGGACCTTCCAGGATGAGGACA	GTTCATCTCGGAGCCTGTAGTG
mucolipin 1	GTCGGTGTCATTCGCTACCTGA	GAACGATCCAGCCACAGAAGCA
mucolipin 2	TACGTCCTGGTCACTATCAGCG	GAGCAAGATGCTGCACACGTCA
TPC1	CCCTGGAGTTACCTCGTGTTTC	GAATGCCGTGACCGAGAAATCG
TPC2	CATCCACCTGTGTCTCTTCACC	GTGAGGTCAGTGCTTCTGGAAG
Nanog	GAACGCCTCATCAATGCCTGCA	GAATCAGGGCTGCCTTGAAGAG
c-kit	GAGTTCCATAGACTCCAGCGTC	AATGAGCAGCGGCGTGAACAGA
